# Electrosensitization assists cell ablation by nanosecond pulsed electric field in 3D cultures

**DOI:** 10.1038/srep23225

**Published:** 2016-03-18

**Authors:** Claudia Muratori, Andrei G. Pakhomov, Shu Xiao, Olga N. Pakhomova

**Affiliations:** 1 Frank Reidy Research Center for Bioelectrics, Old Dominion University, Norfolk, VA 23508, USA; 2 Department of Electrical and Computer Engineering, Old Dominion University, Norfolk, VA 23508, USA

## Abstract

Previous studies reported a delayed increase of sensitivity to electroporation (termed “electrosensitization”) in mammalian cells that had been subjected to electroporation. Electrosensitization facilitated membrane permeabilization and reduced survival in cell suspensions when the electric pulse treatments were split in fractions. The present study was aimed to visualize the effect of sensitization and establish its utility for cell ablation. We used KLN 205 squamous carcinoma cells embedded in an agarose gel and cell spheroids in Matrigel. A local ablation was created by a train of 200 to 600 of 300-ns pulses (50 Hz, 300–600 V) delivered by a two-needle probe with 1-mm inter-electrode distance. In order to facilitate ablation by engaging electrosensitization, the train was split in two identical fractions applied with a 2- to 480-s interval. At 400–600 V (2.9–4.3 kV/cm), the split-dose treatments increased the ablation volume and cell death up to 2–3-fold compared to single-train treatments. Under the conditions tested, the maximum enhancement of ablation was achieved when two fractions were separated by 100 s. The results suggest that engaging electrosensitization may assist *in vivo* cancer ablation by reducing the voltage or number of pulses required, or by enabling larger inter-electrode distances without losing the ablation efficiency.

Irreversible electroporation (IRE) has recently emerged as a new minimally invasive procedure for cancer ablation^1^,^2^. In IRE, tumor cells are killed by applying microsecond duration, high intensity electric pulses (EP) which cause irreparable cell damage. The advantage of IRE over other ablation methods such as radiofrequency heating and cryotherapy include better preservation of extracellular tissue scaffold and the ability to treat tumors adjacent to large blood vessels^3^,^4^,^5^,^6^,^7^.

IRE occurs in a limited range of parameters when the treatment is intense enough to kill cells but not too intense to cause thermal damage. Optimization of exposure parameters to fit these requirements has been a subject of active research. One review^8^ have recently analyzed the safety and efficacy of IRE treatments in 16 clinical studies in 221 patients with advanced malignancies of the liver, pancreas, kidneys, lesser pelvis, lungs and lymph nodes. The studies found IRE safe for use in human and efficient for small tumors including those located near major vessels and bile ducts. However, the efficacy of the treatment decreased with increasing the size of the tumor. Because of the limited voltage output of devices approved for IRE, tumors bigger than 3 cm in diameter require multiple electrode installations which add the complexity to the procedure and make it more invasive.

Recent research has extended IRE protocols to nanosecond pulse durations. Nanosecond electric pulses (nsEP) may impair the barrier function of the cell membrane as well as of endoplasmic reticulum and mitochondrial membranes^9^,^10^,^11^,^12^,^13^,^14^. Downstream effects of membrane permeabilization by nsEP range from transient calcium mobilization^12^,^13^,^15^,^16^ to cell uptake of membrane-impermeable solutes^17^,^18^ and induction of necrosis and apoptosis^9^,^19^,^20^,^21^. These bioeffects may assist IRE outcome. Indeed, the application of high voltage, 300 ns pulses to murine melanomas *in vivo* induced both necrosis and apoptosis, resulting in complete tumor remission^22^,^23^,^24^. In addition to initiating apoptosis in tumor cells, nsEP have been shown to block tumor blood flow^23^.

Recently we found that cells exposed to EP develop a delayed and profound increase in the sensitivity to subsequent EP treatments. The cytotoxic effect of EP could be increased 2–3 fold by splitting a high-rate train into two identical trains with an interval long enough to engage sensitization^25^: The first train made cells more sensitive to the cytotoxic effect of the second train, thereby making the entire treatment more efficient. This effect has been named delayed electrosensitization and has been reported for several cell lines (U937, Jurkat, and CHO), different pulse durations (60 ns–100 μs), and pulse amplitudes (1.8–13.3 kV/cm). Sensitization profoundly enhanced the uptake of propidium dye and of bleomycin^26^ (a cell-impermeable cytotoxic agent used for electrochemotherapy^27^,^28^,^29^). These data suggested that EP permeabilized cell membrane more efficiently in sensitized cells.

Engaging sensitization in target tissue by EP may potentially assist IRE-based therapies. Examples include achieving the same ablation effect at lower voltages and pulse numbers; the creation of larger ablation zones without increasing EP amplitude or number; and increasing the distance between EP-delivering electrodes without losing the ablation efficiency. However, previous studies have demonstrated electrosensitization only in single attached cells and in cell suspensions^25^,^26^. The present work is the first trial of electrosensitization in diverse 3D settings *in vitro*. Three-dimensional culture models have been recently used to study the ablation zone after EP exposure^30^,^31^. For the first time, we visualized and quantified the increase of cell killing efficiency and the enlargement of the affected area when sensitization was evoked by a split-dose (i.e., fractionated) EP delivery. These data provide grounds for animal and human trials of cancer ablation using split-dose EP treatment protocols.

## Materials and Methods

### Cell line and media

Experiments were performed using the KLN 205 cell line (ATCC, Manassas, VA), which is an established model of mouse squamous cell carcinoma^32^. Cells were cultured in Eagle’s Minimum Essential Medium (EMEM) with L-glutamine (ATCC), supplemented with 10% (v/v) fetal bovine serum (Atlanta Biologicals, Norcross, GA), 100 U/ml penicillin and 0.1 mg/ml streptomycin (Mediatech Cellgro, Herdon, VA).

### EP exposure and local electric field simulation

All EP exposures in both cuvettes and 3D cultures were performed at room temperature. Procedures for EP exposure of cells in suspension were similar to previously described^19^,^20^,^25^. Trapezoidal pulses of 300 ns duration from an AVTECH AVOZ-D2-B-ODA generator (AVTECH Electrosystems, Ottawa, Ontario, Canada) were delivered to 1-mm gap electroporation cuvettes (BioSmith, San Diego, CA) via a 50- to 10-Ohm transition module (AVOZ-D2-T, AVTECH Electrosystems) modified into a cuvette holder. To produce pulse trains of predetermined duration at selected repetition rates, the generator was triggered externally from a model S8800 stimulator (Grass Instrument Co., Quincy, MA).

**Figure 1:**
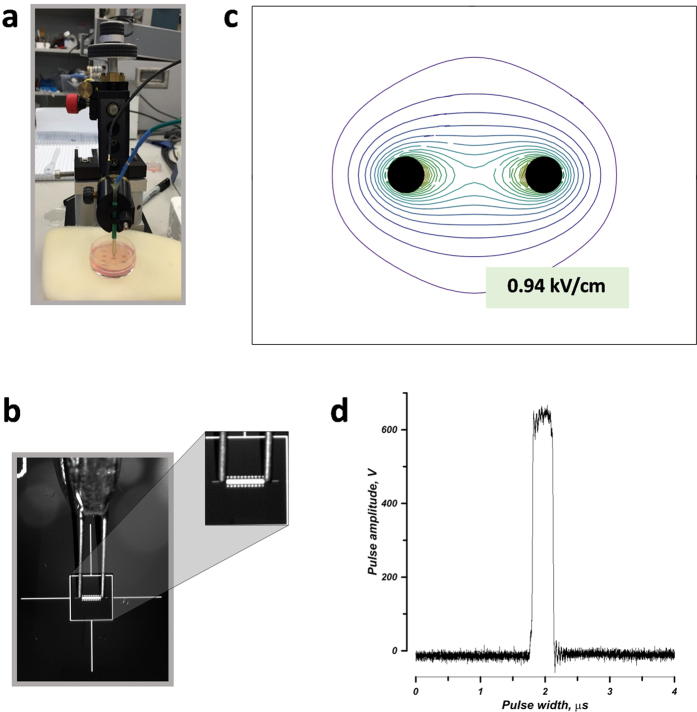
Electroporation in 3D cell cultures. (**a**) A two-needle probe for electric pulse delivery was positioned with a micromanipulator. (**b**) A close-up view of the needles showing 1-mm separation. (**c**) The electric field distribution in the plane perpendicular to the needle electrodes (filled circles). For 600 V applied between the electrodes, the electric field at the outer contour is 0.94 kV/cm; the next contours are drawn at 0.47 kV/cm increments. (**d**) The shape of the electric pulse at 600 V.

EP delivery to cells embedded in an agarose gel or cell spheroids in Matrigel was accomplished by using a custom-made two-needle probe (0.25-mm diameter tungsten rods with 1 mm separation). The probe was mounted in a micromanipulator to enable accurate and steady positioning of the needles within a gel with cells in a 35-mm Petri dish (Fig. 1A,B). For accurate comparison, different EP treatments and sham exposure (no pulses delivered) were performed in the same cell sample, with up to 10 exposures per dish.

The electric field distribution in the plane perpendicular to the needles (Fig. 1C) was determined by a numeric simulation using a finite element Maxwell equations solver Amaze 3D (Field Precision, Albuquerque, NM), as described in detail earlier^33^,^34^. Using the local electric field values, the maximum possible local heating (disregarding any heat losses) was calculated from the adiabatic heat equation^35^. The calculated value for the center of the gap between the needle electrodes was 2 °C per 100 pulses, and the actual temperature rise was even lower due to heat dissipation. In practice, EP trains that were used here to study sensitization effects (up 600 pulses) did not raise the temperature to potentially damaging levels.

The pulse amplitude and shape (Fig. 1D) were monitored in all experiments, using a 500 MHz, 5 GS/s TDS 3052B oscilloscope (Tektronix, Wilsonville, OR, USA).

### EP cytotoxicity in cells exposed in cuvettes

For pulse exposures in cuvettes cells were detached by treatment with 0.5 mg/ml trypsin-EDTA, resuspended at 1.2 × 10^6^ cell/ml in fresh medium, and 100 μl samples of this suspension were aliquoted to electroporation cuvettes for nsEP exposures. All samples remained in cuvettes until all exposures in the experiment were completed. Immediately following the exposures, the samples were diluted with fresh medium to 3 × 10^5^ cells/ml and aliquoted into a 96-well plate, in triplicates at 30 × 10^3^ cell/well and left at 37 °C in the incubator. The next day (22–24 h after the exposure), 10 μl of Presto Blue reagent (Life Technologies, Grand Island, NY) was added to each well and the incubation continued for 15 min at 37 °C. The plate was read with a Synergy 2 microplate reader (BioTek, Winooski, VT), with excitation/emission settings at 530/590 nm. The triplicate data were averaged, corrected for the background, and considered as a single experiment. Data are presented below as a mean +/− SE for *n* independent experiments.

### Three-Dimensional cell cultures

For the 3D cultures we coated the bottom of a 35 mm dish with 2.5 ml of 2.5% low-gelling-temperature agarose (Sigma-Aldrich, St. Louis, MO) in the growth medium. The EP-delivering electrodes left visible traces in the 2.5% agarose layer, which later served as landmarks of EP-treated areas.

For the 3D cultures in agarose, cells were resuspended at 5 × 10^6^ cell/ml in 1% agarose in the growth medium, and 1.5 ml of this suspension was pored over the 2.5% agarose base layer in a 35 mm dish. The samples were then incubated at 4 °C for 5 min to speed up agarose jellification thus avoiding cell sedimentation, and then kept in the incubator for 30 min before EP treatment.

To generate KLN 205 spheroids, the cells were resuspended at 0.5 × 10^6^ cell/ml in Matrigel matrix (Corning, Corning, NY) and 1.2 ml of the suspension was pored over the agarose base layer. Spheroids were grown for 6 days before exposure, in the incubator at 37 °C with 5% CO_2_ in air.

### EP cytotoxicity in 3D cultures, cell imaging and data analysis

Immediately after the EP exposure, 3D cultures were covered with 1.5 ml of media and kept in the incubator until measurements. Because of the suboptimal culture conditions for KLN 205 cells in agarose, 1% agarose cultures where analyzed at 2 h after exposure, whereas spheroid 3D cultures were analyzed the next day after EP treatments.

Dead cells were stained using 4 μg/ml of propidium iodide (PI), (Sigma-Aldrich) in PBS. Thirty minutes before the analysis, the growth medium was replaced with 1 ml of PI solution. In addition to dead cells, in some experiments we also stained viable cells using 3-(4,5-Dimethylthiazol-2-yl)-2,5-diphenyltetrazolium bromide (MTT) (BioAssay Systems, Hayward, CA). Metabolic active cells reduce tetrazolium salts to formazan which accumulate in crystals inside the cells. These crystals, if not solubilized as in standard MTT assay protocols, can be visualized by brightfield transillumination^36^.

Images of the ablation zone were acquired using an Olympus SZX16 fluorescence stereo microscope (Olympus America, Hamden, CT) equipped with a Hamamatsu C9100 EM-CCD camera using a 0.9x, 0.44 NA objective.

**Figure 2:**
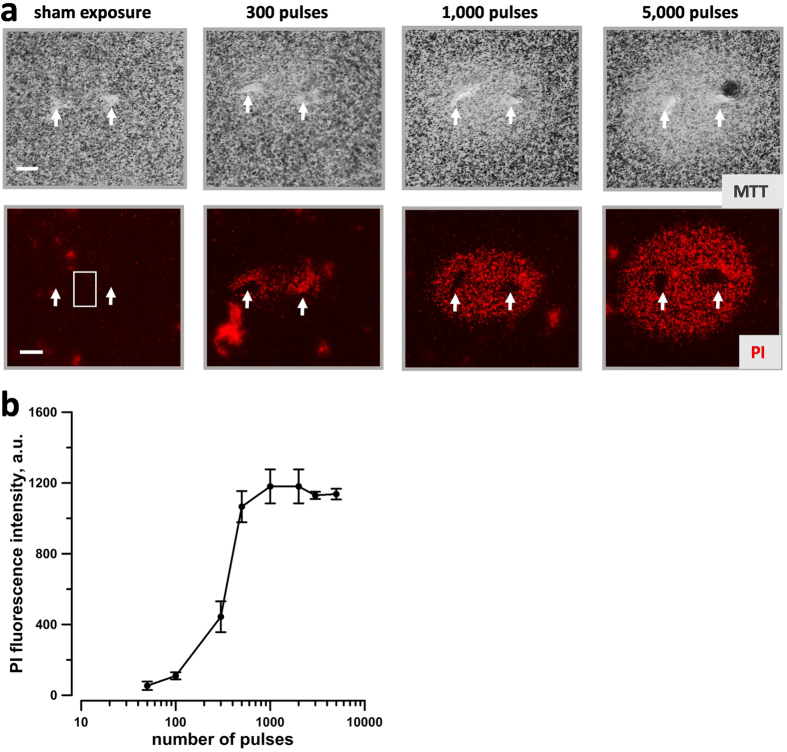
Visualization and quantification of the cytotoxic effect of 300-ns, 600 V electric pulses in agarose gel-embedded cells. (**a**) The ablation area between and around EP-delivering electrodes (arrows) was visualized by the reduction of metabolic MTT conversion into blue formazan (top row, bright field transillumination) and by propidium fluorescence in lethally damaged cells (bottom row). Images were taken 2 hr after the exposure to the indicated number of pulses. Scale bar: 0.5 mm. (**b**) Quantification of the cytotoxic effect by the mean intensity of propidium fluorescence as measured within a box shown in panel (**a**). Mean +/− s.e., n = 3. Note signal saturation at 500–1000 pulses, which indicates killing of 100% of cells within studied region.

Images were quantified with MetaMorph 7.5.2 software (Molecular Devices, Foster City, CA). PI signal in the areas immediately adjacent to the electrodes (where cells experienced the highest electric field gradient and possibly pH changes^37^,^38^) were excluded from the fluorescence quantification. Therefore, in most experiments the PI signal has been quantified within a rectangular region between the electrodes (Fig. 2A). For each image, the fluorescence intensity of the exposed area was corrected for the background fluorescence. Data are presented as mean +/− SE for *n* independent experiments.

### Calibration of the propidium iodide signal

To identify the propidium iodide fluorescence value that corresponded to 100% cell death we exposed KLN 205 embedded in 1% agarose to increasing numbers of 300 ns pulses at 600 V (Fig. 2). At 2 h after the exposure, we stained both viable and dead cells. Significant cell killing was detected after 300 or more pulses, both as a decrease in tetrazolium salt reduction and as an increase in PI uptake (Fig. 2A). To quantify this effect, we measured the PI fluorescence intensity in the region between the electrodes as shown in Fig. 2A. The PI fluorescence intensity reached a plateau at 500–1000 pulses indicating that at these doses 100% of the cells in the quantified area were killed (Fig. 2B). Therefore, in subsequent experiments a parallel control exposure to 3000 pulses was used as a reference point for PI expression that corresponds to 100% cell death. A similar calibration was done for the spheroids exposed in Matrigel and the plateau was reached at 1000–2000 pulses (data not shown).

## Results

### Dose fractionation facilitates killing of KLN 205 cells in suspension

**Figure 3:**
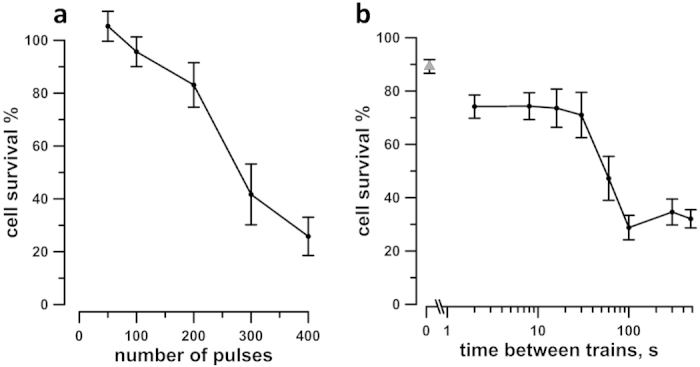
Cytotoxic efficiency of 300 ns, 50 Hz pulses at 6 kV/cm for KLN205 cells in suspension. Exposures were performed in 1-mm gap electroporation cuvettes and cell survival was assessed in 24 hr. (**a**) The effect of pulse number when pulses were delivered in a single train. The survival is in% to sham-exposed parallel control. (**b**) The effect of inter-train interval when 200 pulses were delivered as two 50-Hz trains, 100 pulses each. The inter-train interval of 20 ms (gray triangle) corresponds to a single train treatment with 200 pulses. Mean +/− s.e. for 7 (**a**) or 5 (**b**) independent experiments. Note the sharp decrease of cell survival when the inter-train interval reached about 100 s (**b**), presumably because of sensitization; see text for more details.

Before starting our experiments in 3D cultures we studied the sensitivity to EP and established the conditions to reach maximum electrosensitization in KLN 205 exposed in suspension in electroporation cuvettes. Figure 3A shows the effect of increasing pulse number on cell survival whereas other treatment parameters were fixed (one train at 50 Hz, 300 ns pulse duration, 6 kV/cm). A dose of 200 pulses, which caused the smallest significant decrease of viability (by 10–20%) as a single train, was chosen to study the effect of dose fractionation. Cells were exposed to either a single train of 200 pulses, or two trains of 100 pulses each, with the inter-train intervals from 2 to 480 s (Fig. 3B). Maximum facilitation of the EP cytotoxic effect was achieved at the interval of 100 s, when the cell survival fell 3 times below the no-sensitization control. The same data can also be viewed as a 7-fold increase of cell killing (from approx. 10% to 70% when applying single- and split-dose treatments). Longer intervals of 300 and 480 s showed the effect similar to 100 s. The number of pulses and the inter-train interval established in these experiments have been used in most of the following experiments in 3D cultures.

### Fractionated EP treatments increase the ablation volume and cell killing in agarose-embedded cells

**Figure 4:**
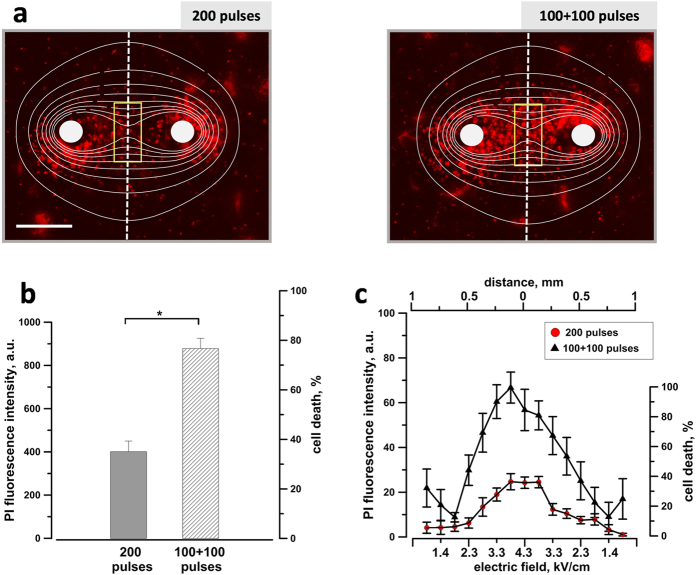
Exposure fractionation increases cytotoxicity in agarose embedded cells. KLN 205 cells were exposed to either a single dose of 200 pulses (300 ns, 600 V, 50 Hz) or two doses of 100 pulses each with 100 s interval. Panel (**a**) shows the electric field map superimposed on representative PI fluorescence images of each sample. Scale bar: 0.5 mm. The quantification in (**b**) shows the PI uptake (left Y-axis) and the percentage of cell death (right Y-axis) measured within the yellow box. Panel (**c**) shows PI fluorescence and percentage of cell death within 15 regions of interest drawn along the dashed line as function of the local electric field. Mean +/− s.e., n = 5. *p < 0.001.

To study the effect of dose fractionation on the ablation zone we set up a 3D culture in agarose where KLN 205 cells are exposed to EP in a solid phase and the ablation zone is visualized and quantified by PI uptake by the dead cells (Fig. 4). When a single train of 200 pulses at 600 V was applied, cell death was observed mainly around the electrodes. Conversely, by splitting the exposure in two trains of 100 pulses each with 100 s interval, the PI signal significantly increased, especially in the area between the electrodes (Fig. 4A). Compared to a single dose, the split-dose treatment caused up to two-fold increase in the PI uptake (as averaged in a region between the electrodes, (Fig. 4B) and increased the lethality from 35–40 to 75–80%. Moreover, by overlaying the fluorescence images with the electric field map we measured the efficiency of cell killing as a function of the local electric field. Fifteen regions along the dashed line shown in (Fig. 4A) and corresponding to different electric field strengths were utilized to measure the PI uptake and cell death rate (Fig. 4C). At the highest field strength (4.3 kV/cm), split dose treatments caused up to 3-fold increase in PI uptake over the effect of single trains, reaching 100% cell killing. At 3.3 and 2.3 kV/cm sensitization caused 70% and 40% cell death, respectively, whereas a single train killed only 15–20% of cells. Our data indicate that, for 300 ns pulse duration, sensitization occurs when the electric field exceeds 1.9 kV/cm, and that engaging electrosensitization increases cell death across a wide range of electric field values.

**Figure 5:**
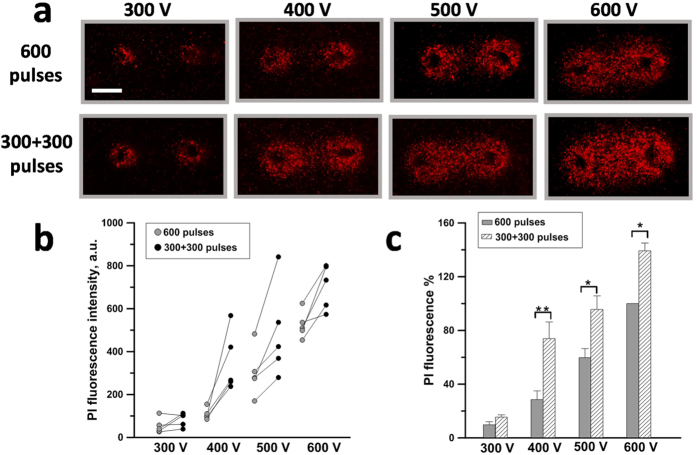
Engagement of sensitization at different applied voltages. KLN 205 seeded in 1% agarose were exposed to either a single train of 600 pulses (300 ns, 50 Hz) or two trains of 300 pulses each. The pulse amplitude was varied from 300 to 600 V. (**a**) representative fluorescence images from one experiment. Scale bar: 0.5 mm. Panel (**b**) shows for each experiment the PI fluorescence value as a function of the applied voltage. The lines connecting the symbols identify samples from single and split dose exposure belonging to the same experiment. In (**c**), the PI uptake is in % to the area of the same gel exposed to a single train of 600 pulses at 600 V. Mean +/− s.e., n = 5, *p < 0.05, **p < 0.01. Split dose treatments were more efficient at 400, 500 and 600 V.

This finding prompted us to investigate the efficiency of split dose treatments at lower applied voltages which consequently required a higher number of pulses (Fig. 5). Six hundred pulses (300 ns, 50 Hz) were delivered either as a single train or as two trains with 100 s interval and the voltage applied was varied from 300 to 600 V. Fig. 5A shows for a representative experiment that fractionated treatments produced a larger PI-positive region. Fig. 5B shows the PI fluorescence as a function of the applied voltage for all five experiments performed. The lines connecting the different symbols identify the single and fractionated dose samples from the same experiment. In all the experiments at 400, 500, and 600 V, the fractionated exposure was more efficient. Maximum sensitization effect was seen at 400 V (2.9 kV/cm in the center between the electrodes) when the PI signal increased 2.5 times (Fig. 5C). Fractionated doses at 400 and 500 V were as efficient as single dose at the next higher pulse amplitude (500–600 V).

**Figure 6:**
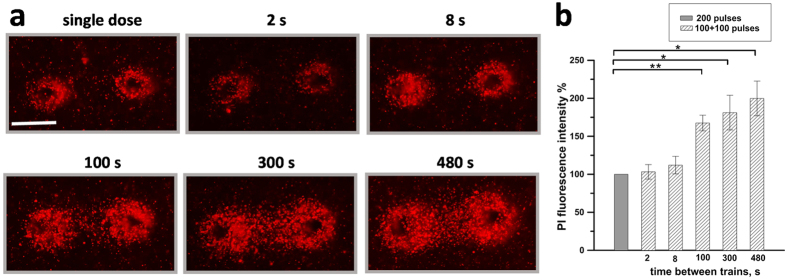
Inter-train interval as short as 100 s causes maximum sensitization in cells exposed in 1% agarose. KLN 205 cells were exposed to either a single train of 200 pulses (300 ns, 600 V, 50 Hz) or two trains of 100 pulses each. The interval between the two trains was varied from 2 to 480 s (as indicated in legends). PI uptake was measured 2 hr after the exposure. Scale bar: 0.5 mm. In (**b**) the PI fluorescence value is in % to the area of the same gel exposed to a single train of 200 pulses. Mean +/− s.e., n = 4. *p < 0.05, **p < 0.01.

To explore the time course of sensitization in a 3D setting, agarose-embedded cells were exposed either to a single train of 200 pulses, or to split-dose treatments (100 + 100 pulses) at inter-train intervals from 2 to 480 s. A representative experiment in Fig. 6A shows that fractionation enhanced PI uptake when the inter-train interval reached 100 s. However, no significant further increase in cell death was observed with longer intervals of 300 and 480 s (Fig. 6B). Noteworthy, this time course was similar to the data for cells in suspension (Fig. 3B), with 100 s being the shortest interval to achieve the maximum facilitation of cell killing by engaging electrosensitization.

### Exposure fractionation increases cell death in KLN 205 spheroids

**Figure 7:**
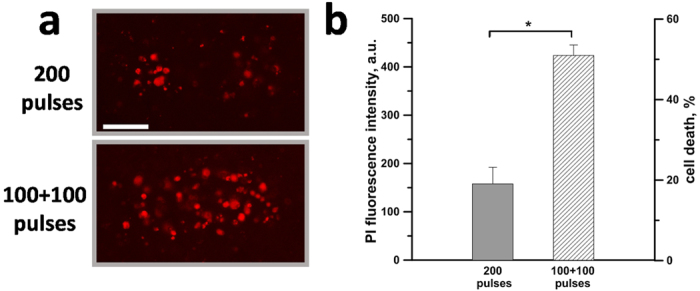
Split dose treatments increase cell death in KLN 205 spheroids. (**a**) KLN 205 spheroids grown in Matrigel were exposed to either 200 pulses or two trains of 100 pulses each. PI uptake was measured 24 hr after treatment. Scale bar: 0.5 mm. Panel (**b**) shows the PI uptake (left *y*-axis) and the percentage of cell death (right *y*-axis). Mean +/− s.e., n = 4. *p < 0.01.

Finally, we investigated whether electrosensitization occurs also in spheroids which are known to respond in a manner more similar to that observed in tumors *in vivo*^39^. KLN 205 spheroids grown in Matrigel were exposed to 200 pulses (300 ns, 6 kV/cm, 50 Hz) either as single dose or as two fractions with 100 s interval and the PI uptake was measured 24 h after the exposure. Figure 7A shows that EP fractionation enhanced cell death in spheroids. The data in Fig. 7B show a 2.6-fold increase in PI uptake in sensitized samples, which corresponded to the increase of cell death rate from 20% to 50%. Overall, our results show that fractionated treatments are also more efficient in multicellular spheroids.

## Discussion

This study expands the range of known experimental conditions when fractionated delivery of EP can significantly increase their cytotoxic effect. To date, higher efficiency of fractionated EP treatments was established in multiple cell lines (CHO, B16, U937, Jurkat, N1-S1), for EP durations ranging from 60 ns to 100 μs, and for diverse culture conditions (suspensions, substrate-attached cells, cells embedded in agarose gel, and cell spheroids)^25^,^26^,^40^,^41^,^42^. Such universality further helps to exclude possible impact of other factors which could affect cytotoxicity (such as cell sedimentation and random rotation in suspensions)^43^. As discussed earlier^25^,^40^, gradual resealing of the cell membrane between sequential pulses (which was proposed as a simple explanation of stronger effects at lower pulse repetition rates)^43^ does not hold for split-dose protocols with high pulse rates within each train. This explanation also contradicts the experimental evidence that the second high-rate EP train causes more membrane damage than the identical first one (e.g., see Fig. 4 in Gianulis *et al.*)^41^. The present body of knowledge unequivocally identifies the delayed electrosensitization as a biological phenomenon responsible for the high efficiency of fractionated EP treatments, albeit the underlying physiological mechanisms remain elusive. We have suggested that sensitization could result from 1) EP-induced colloid-osmotic cell swelling, 2) influx of Ca^2+^ and its downstream effects, 3) oxidative damage to the membrane, 4) cytoskeleton disruption, and 5) ATP leakage and depletion. However, none of these mechanisms has been experimentally established as a cause of sensitization. As a matter of fact, even the broader topic of how intense nsEP treatments lead to cell death and how this process is affected by pulse parameters, environment, and cell physiology remains far from being understood, despite the intense ongoing research^9^,^19^,^20^,^21^,^27^,^44^,^45^,^46^,^47^,^48^,^49^,^50^,^51^,^52^,^53^.

In parallel with the mechanistic studies, the research into practical applications of nsEP for tumor ablation in animal models and even in humans is progressing fast^22^,^23^,^27^,^44^,^54^,^55^. The engagement of electrosensitization by applying split-dose pulse delivery protocols may assist tumor ablation in various ways, such as achieving the same therapeutic effect with fewer pulses or at lower voltages, reducing thermal effects, or further spreading of electrodes to better encompass the tumor. This study was the first to demonstrate the benefits of sensitization in 3D models, and to visualize the enlargement of the affected area and greater extent of cell killing. Importantly, the full benefit of sensitization was already achieved with the inter-train interval of just 100 s, which is certainly more practical for clinical treatments than the earlier reported interval of 5 min^25^,^26^,^40^. It remains to be established if this shorter time interval is characteristic for the specific cell type, or EP exposure conditions, or both. Cells in suspension and embedded in matrices responded to fractionated treatments very similarly, suggesting that sensitization should occur *in vivo* as well. Therefore, our ongoing work aims at demonstrating the benefit of split dose treatments for tumor ablation *in vivo* in mice as well as at establishing the physiological mechanisms of electrosensitization.

## Additional Information

**How to cite this article**: Muratori, C. *et al.* Electrosensitization assists cell ablation by nanosecond pulsed electric field in 3D cultures. *Sci. Rep.* **6**, 23225; doi: 10.1038/srep23225 (2016).
